# A Pan-Cancer Landscape of ABCG2 across Human Cancers: Friend or Foe?

**DOI:** 10.3390/ijms232415955

**Published:** 2022-12-15

**Authors:** Chen Lyu, Lili Wang, Birgit Stadlbauer, Alexander Buchner, Heike Pohla

**Affiliations:** 1 Tumor Immunology Laboratory, LIFE Center, LMU Klinikum, University Munich, 82152 Planegg, Germany; 2 Department of Urology, LMU Klinikum, University Munich, 81377 Munich, Germany

**Keywords:** ABCG2, pan-cancer, prognosis, cancer stem cell, genetic alteration

## Abstract

Emerging evidence from research or clinical studies reported that ABCG2 (ATP-binding cassette sub-family G member 2) interrelates with multidrug resistance (MDR) development in cancers. However, no comprehensive pan-cancer analysis is available at present. Therefore, we explore multiple databases, such as TCGA to investigate the potential therapeutic roles of ABCG2 across 33 different tumors. ABCG2 is expressed on a lower level in most cancers and shows a protective effect. For example, a lower expression level of ABCG2 was detrimental to the survival of adrenocortical carcinoma (TCGA-ACC), glioblastoma multiforme (GBM), and kidney renal clear cell carcinoma (KIRC) patients. Distinct associations exist between ABCG2 expression and stemness scores, microenvironmental scores, microsatellite instability (MSI), and tumor mutational burden (TMB) of tumor patients. We observed a significant positive correlation between the ABCG2 mutation site and prognosis in uterine corpus endometrial carcinoma (UCEC) patients. Moreover, transmembrane transporter activity and hormone biosynthetic-associated functions were found to be involved in the functionality of ABCG2 and its related genes. The cDNAs of cancer cell lines were collected to detect exon mutation sequences and to analyze ABCG2 mRNA expression. The mRNA expression level of ABCG2 showed a significant difference among spheres and drug-resistant cancer cell lines compared with their corresponding adherent cancer cell lines in six types of cancer. This pan-cancer study provides, for the first time, a comprehensive understanding of the multifunctionality of ABCG2 and unveils further details of the potential therapeutic role of ABCG2 in pan-cancer.

## 1. Introduction

ABCG2, known as breast cancer resistance protein (BCRP), was described as the second member of the G subfamily and a subfamily in the ATP-binding cassette (ABC) family proteins, which are expressed in many tissues, such as intestine, liver, kidney, and cancerous tissue [1]. Several studies have revealed that ABCG2 as a biomarker of cancer stem-like cell (CSC) populations can limit the efficacy of chemotherapy and is a target for clinical interventions during the period of tumor relapse [2,3]. In cancer therapy, inhibition of ABCG2 has emerged as a new treatment strategy that is believed to increase chemotherapeutic drug bioavailability and to overcome drug resistance [4].

The structural feature of ABCG2 helps us to further understand drug efflux independent contributions of ABCG2 concerning polydrug recognition and transport.

ABCG2 domain structure is a dimer of two protein chains each consisting of a catalytic nucleotide binding domain (NBD) and a transmembrane domain with at least six membrane spanning α-helices (TMD) [5]. TMD carries the substrate binding site and represents the translocation pathway for substrate, while NBD is responsible for ATP binding and ATP hydrolysis [6]. If a physiological substrate, for example, the molecule estrone-3-sulfate (E1S), is bound in the cavity of the TMD, NBDs can face away from each other and help to identify key residues contributing to substrate recognition. Then, two ATP molecules bind and induce a conformational change of the dimerization of the NBDs that stimulates the sign to close the cavity, thereby leaving no space for other substrate binding. On the extracellular side, the extracellular loop has rotated away from TMD, opening up a smaller cavity so that endogenous substrates can be transported. When the second molecule of ATP is hydrolyzed, the conformational change will be reset, and substrate and ATP can bind again allowing the process to repeat [6]. Moreover, ABCG2 can also remove exogenous substrates, such as a variety of xenobiotics and pharmaceuticals, to limit drug delivery to the brain, thereby strongly influencing drug resistance and chemotherapeutic intervention [7]. Binding of drugs can induce a conformational switch of the transporter, which is observed for several anticancer drugs (imatinib, mitoxantrone, or SN38) with different effects [8].

While the role of ABCG2 in multidrug resistance is widely recognized, the dysfunction of ABCG2 gene can be induced by other factors, such as genetic polymorphisms, which have been proven in several diseases, such as hyperuricemia and hypertension in humans [9]. For example, the common ABCG2 variant-Q141K impairs the stability of NBD, which may affect the pharmacokinetic profile of several drugs in the clinic [10,11]. Several other variants (G406R, F431L, S441N, P480L, F489L, M515R, L525R, A528T, and T542A) located within the transmembrane region of ABCG2 have been shown to affect the activity of the protein in vitro [12]. For non-small cell lung cancer (NSCLC), variant genotypes of ABCG2 rs3114020 were found to be associated with a significantly increased risk of death (*p* < 0.001), and the rs1871744 genotype was significantly associated with poor response, suggesting that ABCG2 mutation patterns can be an independent risk factor for the prognosis of NSCLC [13]. However, it remains unclear, which of the ABCG2 mutation sites are found in different types of cancers and how it might influence the drug–ABCG2 interactions. Therefore, a detailed understanding of the mechanism and genetic polymorphisms of ABCG2 could drive the development of the bioavailability of the desired drug, minimize multidrug resistance, and promote more effective therapeutic strategies.

Our study carried out, for the first time, a pan-cancer analysis of ABCG2 based on The Cancer Genome Atlas (TCGA) (https://www.cancer.gov/tcga) to explore the multifunctional role of ABCG2 in different types of cancer. We also investigated gene expression, genetic alteration, stemness scores, microenvironmental scores, relevant immune checkpoint genes, and cellular function of ABCG2 to reveal the potential molecular pathogenesis of multiple cancers, challenging the pathogenic role of ABCG2 as a reference gene for cancers and presenting a potential prognosis biomarker and immunotherapeutic target.

## 2. Results

### 2.1. ABCG2 Expression in Human Cancers

**Figure 1:**
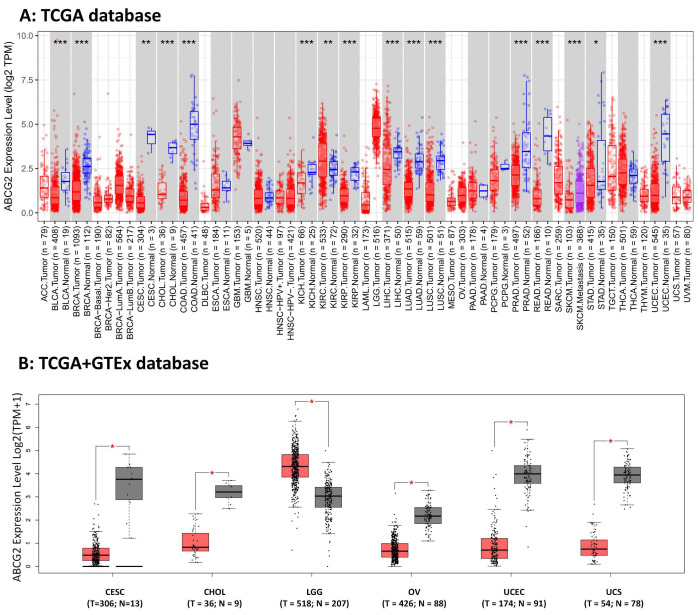

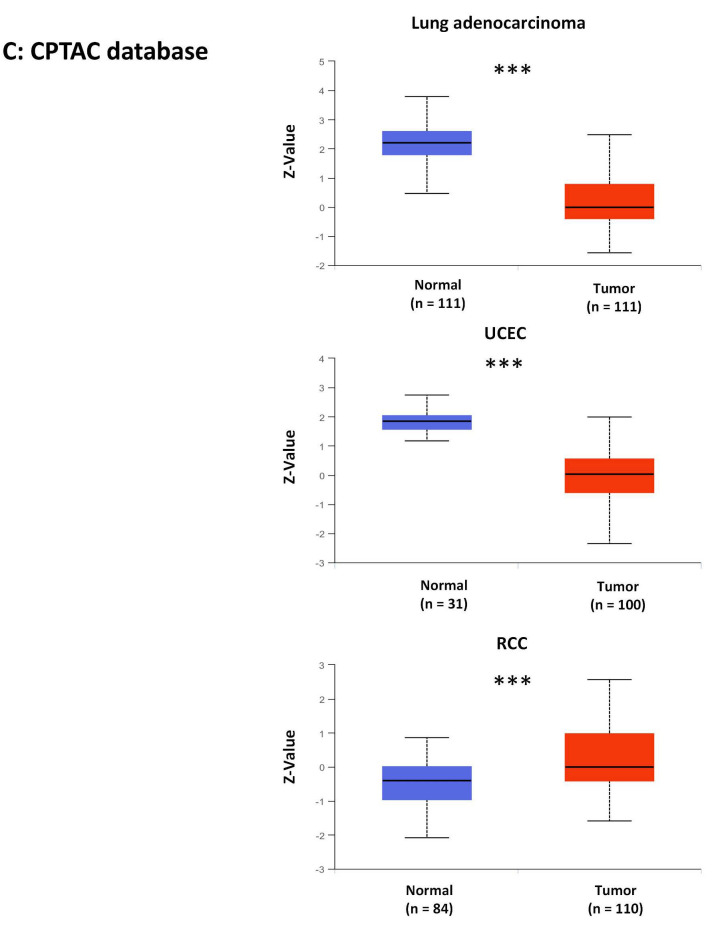
The ABCG2 expression data were obtained from various databases: (**A**) Data collected from TCGA database and analyzed by the TIMER2.0 database. Samples with gray backgrounds represent both tumor (red) and normal tissue (blue) samples and one metastasis (purple), which can be compared statistically. Samples with white backgrounds represent only tumor samples, which cannot be compared statistically because other types of tissue samples were not available (* *p* < 0.05; ** *p* < 0.01; *** *p* < 0.001). (**B**) We used normal tissue data from the Genotype-Tissue Expression (GTEx) database as controls for comparisons with the corresponding data from The Cancer Genome Atlas (TCGA) project. The results are presented as a box plot (* *p* < 0.05). Tumor samples in red and normal samples in black. (**C**) Expression levels of ABCG2 protein were also compared between tumor tissue (red) and normal tissue (blue) in LUAD (lung adenocarcinoma), UCEC (uterine corpus endometrial carcinoma), and RCC (clear cell renal cell carcinoma (RCC)) based on the CPTAC dataset (*** *p* < 0.001).

To determine the effects of ABCG2 on cancer in humans, we used TIMER2.0 to explore ABCG2 expression in various types of cancer from the TCGA database. As demonstrated in (Figure 1A), expression differences of ABCG2 between tumor and normal tissues were found in bladder urothelial carcinoma (BLCA), breast invasive carcinoma (BRCA), cervical squamous cell carcinoma and endocervical adenocarcinoma (CESC), cholangiocarcinoma (CHOL), colon adenocarcinoma (COAD), kidney chromophobe (KICH), kidney renal clear cell carcinoma (KIRC), kidney renal papillary cell carcinoma (KIRP), liver hepatocellular carcinoma (LIHC), lung adenocarcinoma (LUAD), lung squamous cell carcinoma (LUSC), prostate adenocarcinoma (PRAD), rectum adenocarcinoma (READ), skin cutaneous melanoma (SKCM), stomach adenocarcinoma (STAD), and uterine corpus endometrial carcinoma (UCEC) (*p* < 0.05). The expression analysis via TIMER2.0 indicates that different cancers and specific cancer subtypes affect the ABCG2 gene expression status. For some tumors, not enough normal tissue sample was available in the TCGA database (those tumors are shown with a white background in Figure 1A). Then, GTEx dataset was used as control for the normal tissues, and we evaluated the ABCG2 expression differences between tumor and normal tissues of CESC, CHOL, brain lower grade glioma (LGG), ovarian serous cystadenocarcinoma (OV), UCEC, and uterine carcinosarcoma (UCS) (*p* < 0.05, Figure 1B).

The Clinical Proteomic Tumor Analysis Consortium (CPTAC) integrates genomic and proteomic data to identify and describe all proteins within tumor and normal tissues and explores candidate proteins that can be used as tumor biomarkers. Available data from the CPTAC dataset indicate that ABCG2 protein expression was lower in UCEC and LUAD than in normal tissues; Figure 1C and the opposite was shown in RCC.

**Figure 2:**
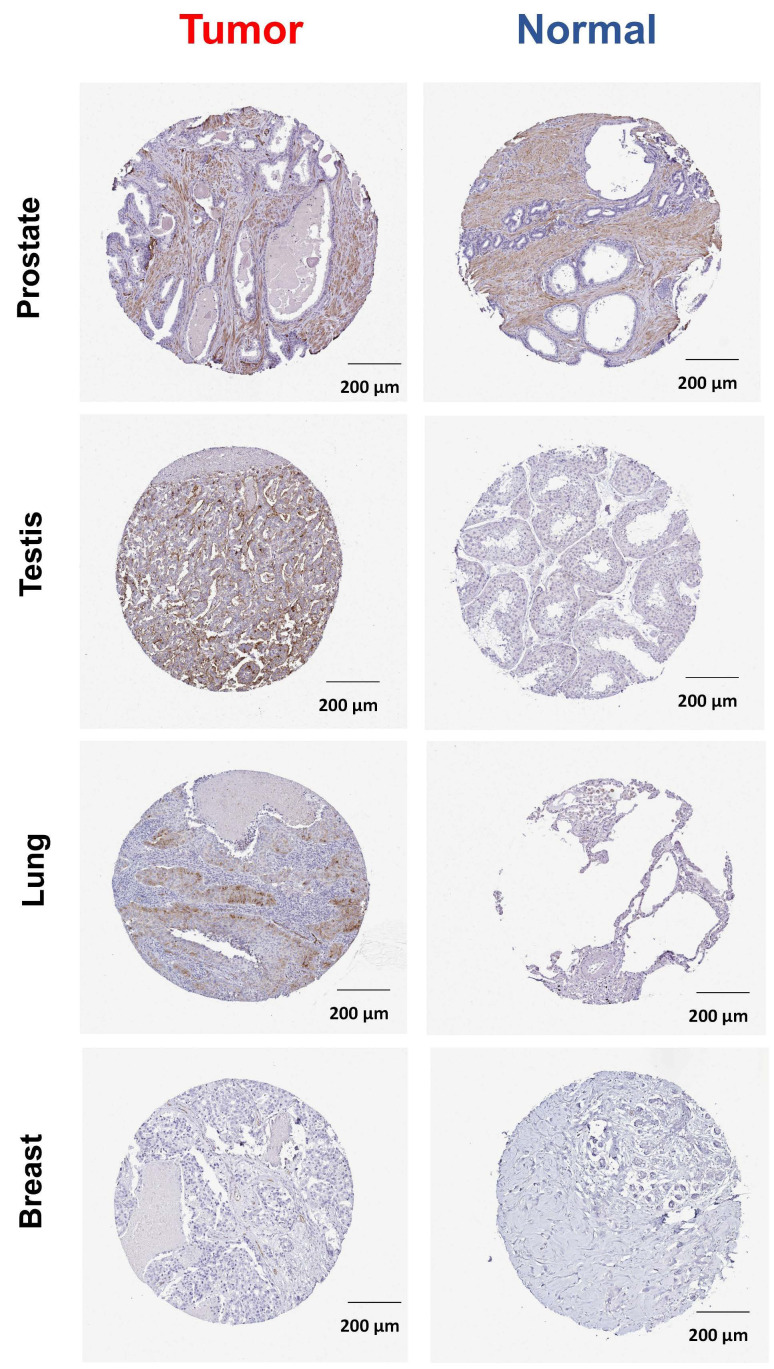
ABCG2 protein expression was shown in immunohistological sections of breast (T: HPA054719 antibody; N: HPA054719 antibody), lung (T: HPA054719 antibody; N: HPA054719 antibody), testis (T: HPA054719 antibody; N: HPA054719 antibody), and prostate (T: HPA054719 antibody; N: HPA054719 antibody), obtained from the Human Protein Atlas database.

The ABCG2 protein expression level between normal samples and breast, lung, testis, and prostate cancer samples was validated using The Human Protein Atlas. The results indicate that the expression level of ABCG2 was markedly increased in normal prostate tissue compared with other normal tissues. For testis, tumor tissue revealed a higher ABCG2 expression compared to the normal tissue (Figure 2).

### 2.2. ABCG2 as a Prognostic Factor in Human Cancers

**Figure 3:**
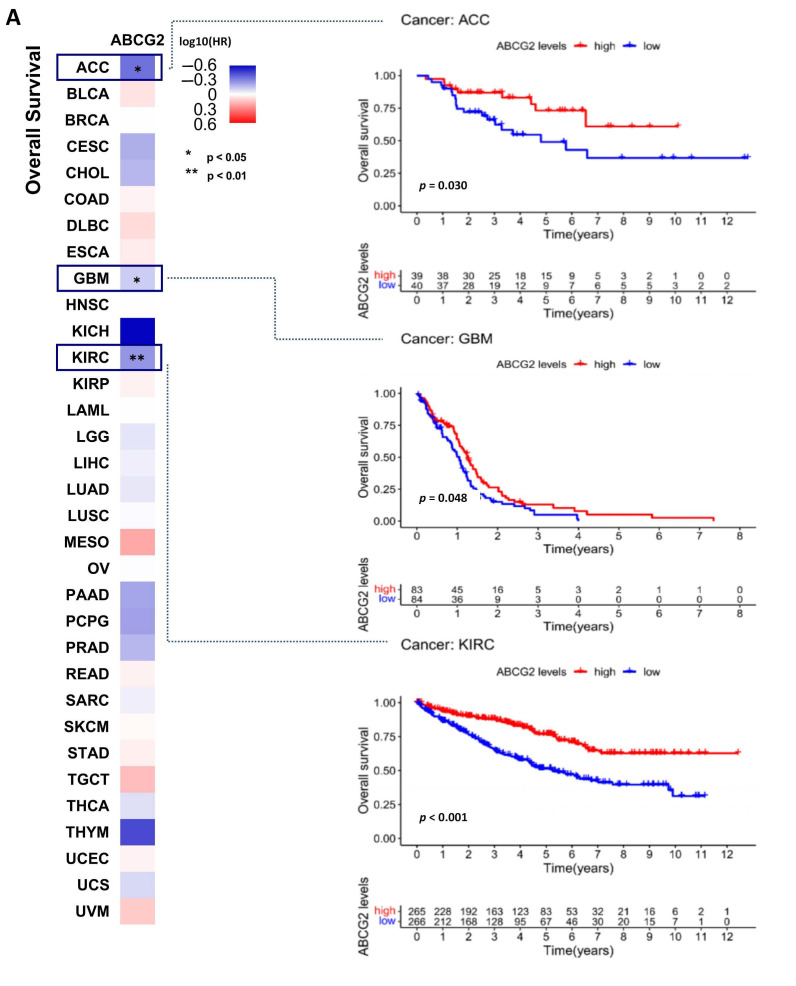

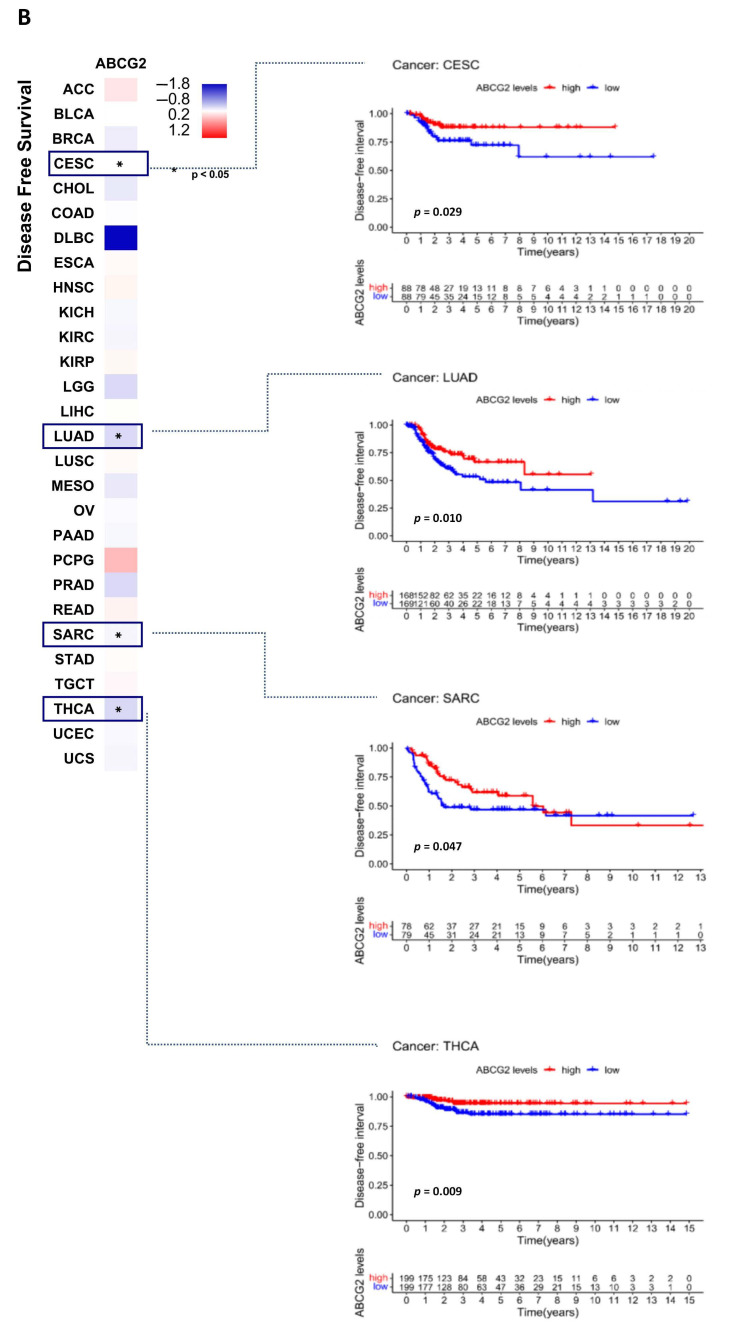
Correlation between ABCG2 gene expression, overall survival, and disease-free survival for different cancer types: (**A**,**B**) The pan-cancer survival maps with significant results are given. For the Kaplan–Meier survival analysis, patients were grouped into high and low ABCG2 expression scores determined by the comparison with the median by log-rank test.

We divided the cancer types into high-expression and low-expression groups according to the expression levels of ABCG2 and investigated the correlation of ABCG2 expression with the prognosis of patients within different tumors. As shown in Figure 3A, analysis of overall survival (OS) demonstrates that a low expression level of ABCG2 was linked to poor prognosis for the cancer types of adrenocortical carcinoma (ACC) (*p* = 0.030), glioblastoma multiforme (GBM) (*p* = 0.048), and KIRC (*p* < 0.001) within the TCGA project. Analysis of disease-free survival (DFS) (Figure 3B) shows a correlation between low ABCG2 expression and poor prognosis for CESC (*p* = 0.029), LUAED (*p* = 0.010), sarcoma (SARC) (*p* = 0.047), and thyroid carcinoma (THCA) (*p* = 0.009).

**Figure 4:**
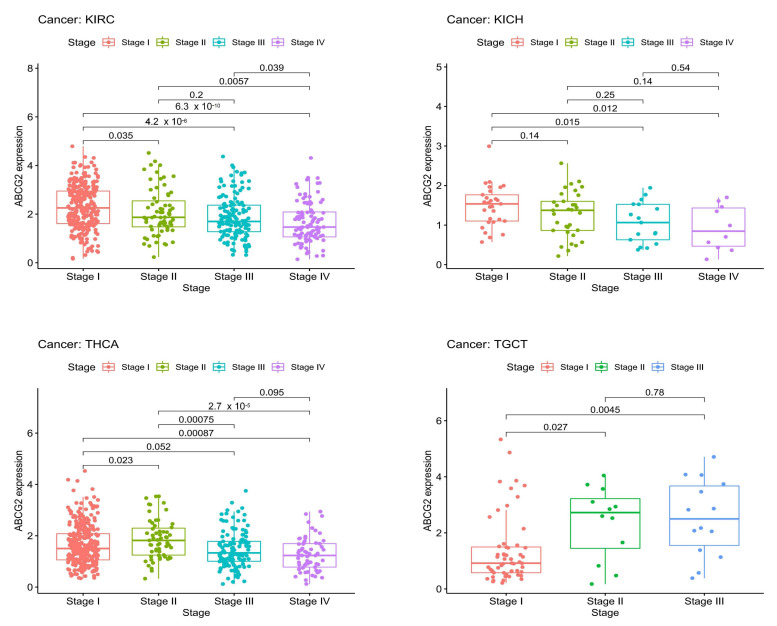
Correlation between ABCG2 gene expression and the pathological stages of different cancer types. The pathological stages (stages I to IV) with significant results are shown.

**Figure 5:**
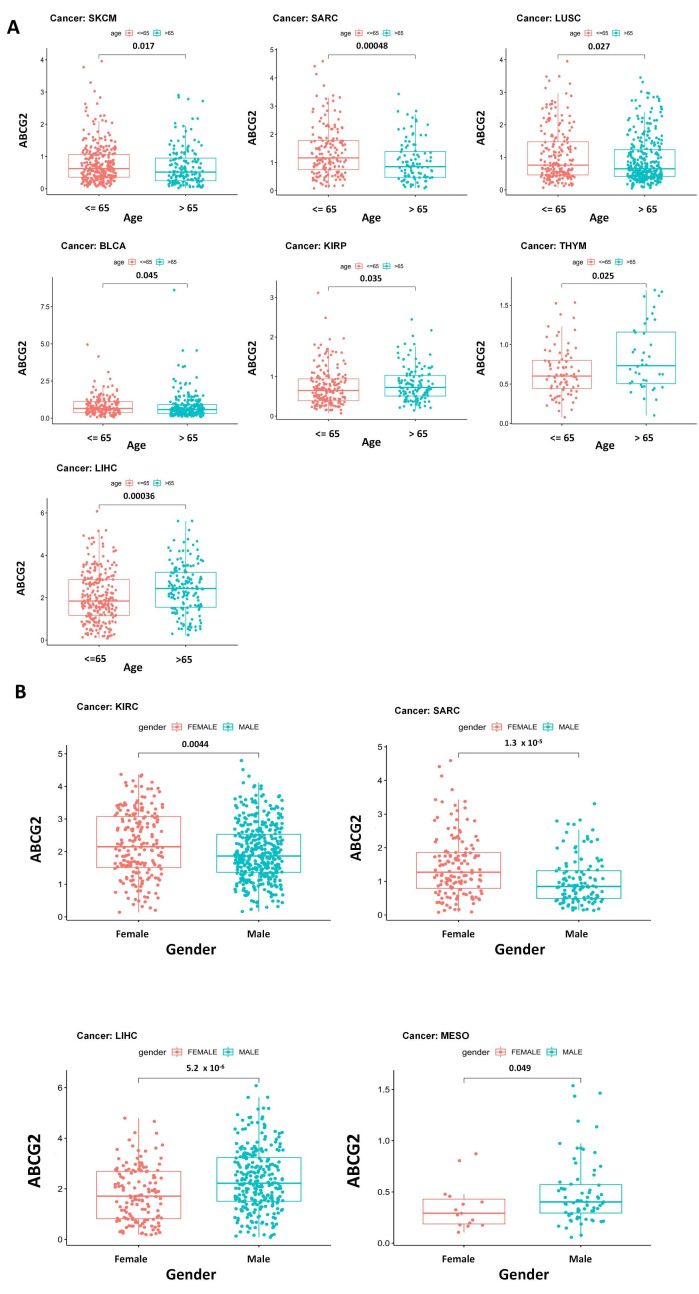
Correlation between ABCG2 gene expression and the clinicopathological characteristics of different cancer types: (**A**) age (≤65 and >65) and (**B**) gender (female and male) with significant results are shown.

Moreover, significant correlations between pathological stages, age, gender, and ABCG2 expression are shown in Figure 4 and Figure 5A,B. For the pathological stage, significant correlations emerge among KICH, KIRC, testicular germ cell tumors (TGCT), and THCA (Figure 4), of which KIRC, THCA, and KICH show ascending tendency with increasing expression of ABCG2, and TGCT shows the opposite trend. Higher expression of ABCG2 was observed in the patient group ≤ 65 years for LUSC, SARC, SKCM, and BLCA, whereas for thymoma (THYM), KIRP, and LIHC in the group > 65 years (Figure 5A). There are also clear differences between the gender groups. For KIRC and SARC, female patients show higher ABCG2 expression, in contrast to mesothelioma (MESO) and LIHC, where male patients show higher ABCG2 expression (Figure 5B).

### 2.3. The Correlation between ABCG2 Expression and RNAss, DNAss, ImmuneScore, and StromalScore

**Figure 6:**
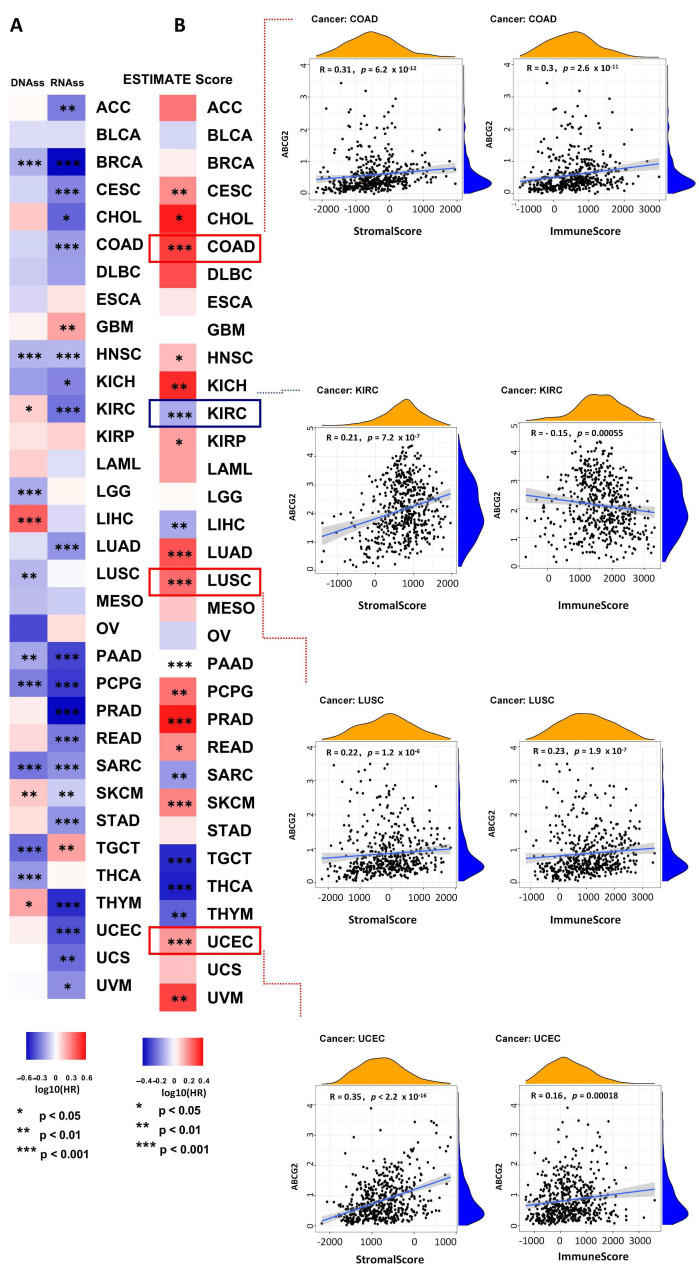
Correlation between ABCG2 gene expression, stemness scores, and microenvironmental scores based on the TCGA database: (**A**) Correlation of ABCG2 expression with the RNA and DNA stemness scores; (**B**) the ESTIMATE score map was analyzed and the StromalScore and the ImmuneScore for COAD, KIRC, LUSC, and UCEC with *p* < 0.001 are shown * *p* < 0.05, ** *p* < 0.01, *** *p* < 0.001. Blue box (downregulation), red box (upregulation).

ABCG2 correlates with drug resistance in several types of cancer possibly due to its expression in CSCs. Tumor stemness can be measured using the RNA stemness score (RNAss) based on mRNA expression and using the DNA stemness score (DNAss) based on DNA methylation pattern. The correlation between ABCG2 expression and tumor stemness measured by RNAss and DNAss was investigated. Interestingly, the results show that ABCG2 is negatively correlated to most types of cancer concerning RNAss and DNAss. Notably, ABCG2 expression shows positive correlation with DNAss but a negative correlation with RNAss in THYM, KIRC, and SKCM, and the opposite tendency is shown in TGCT (Figure 6A). These contradictory results suggest that RNAss and DNAss may identify distinct cancerous cell populations characterized by different features or degrees of stemness in different cancers. ImmuneScore and StromalScore represent newly developed algorithms that take advantage of the unique properties of the transcriptional profiles of cancerous tissues to infer tumor cellularity as well as the various infiltrating normal cells. For this, the ESTIMATE scores can be applied to predict tumor purity, which was calculated by the StromalScore and the ImmuneScore [14]. Specific gene expression signatures of stromal and immune cells can be used to develop a prognosis stratification in cancer. In this study, ABCG2 expression shows a positive correlation with the ImmuneScore and the StromalScore in most types of cancer (Figure 6B). For TGCT, THCA, and THYM, a stronger negative correlation was observed for the expression of ABCG2 and the ImmuneScore and StromalScore (*p* < 0.001). For KIRC patients, ABCG2 expression shows an opposite trend between the ImmuneScore and the StromalScore (*p* < 0.001), of which a positive correlation is observed with the StromalScore and a negative correlation with the ImmuneScore. These results confirm the important role of ABCG2 in the microenvironmental processes.

### 2.4. The Correlation between ABCG2 Expression and TMB, MSI, Genetic Alteration Analysis

**Figure 7:**
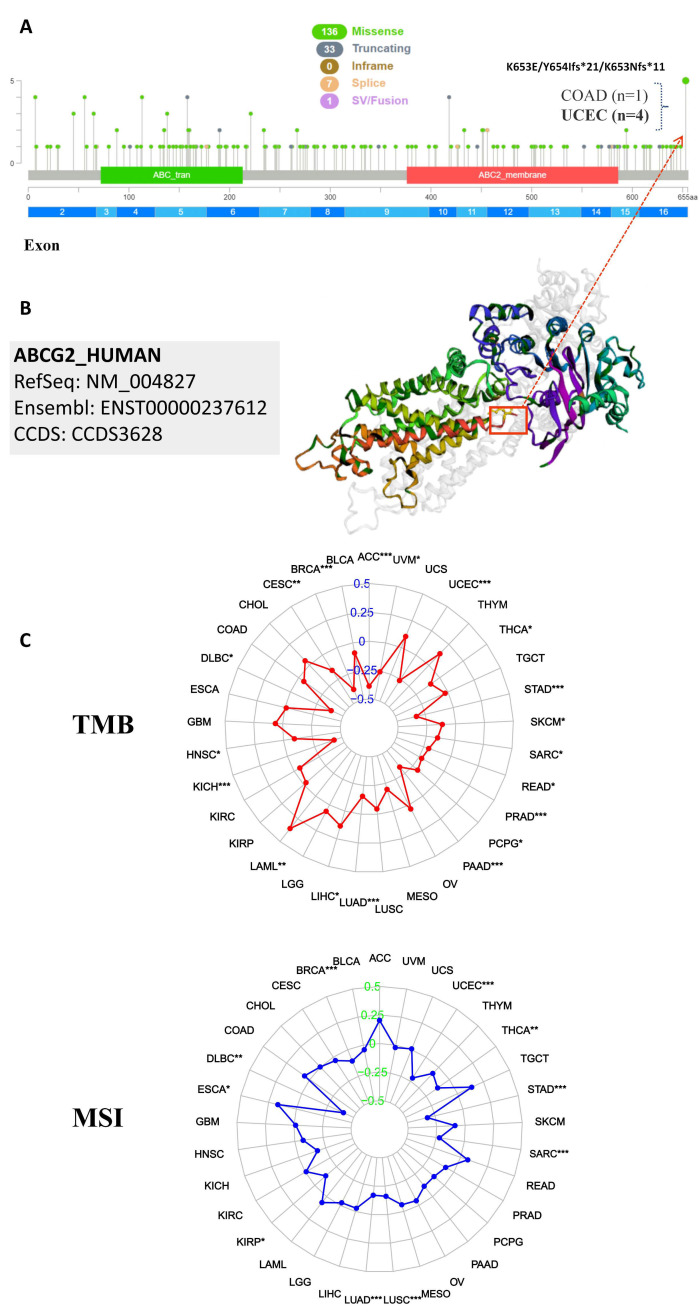
Mutation features of ABCG2 in different tumors: (**A**,**B**) The mutation features of ABCG2 for the TCGA tumors were analyzed using the cBioPortal tool. The alterated mutation sites, the mutation site with the highest frequency (K653E/Y654Ifs*21/K653Nfs*11) (four cases for UCEC and one case for COAD), and the 3D structure of ABCG2 are displayed. (**C**) The radar charts illustrated the association between TMB or MSI and ABCG2 gene expression in different cancers. The red and blue curve represent the correlation coefficient, and the blue and green value represent the range. * *p* < 0.05, ** *p* < 0.01, *** *p* < 0.001.

**Figure 8:**
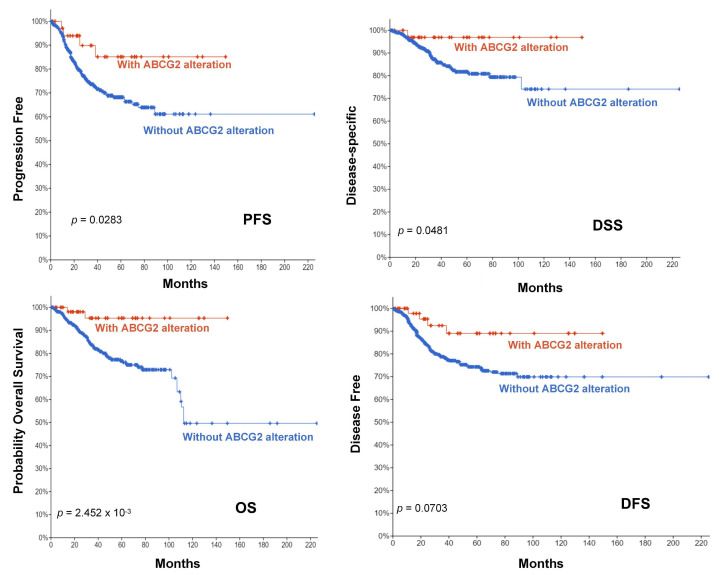
The potential correlation between the mutation site and the survival patterns. The potential correlation between this mutation site and progression-free survival (PFS) (n = 528), overall survival (OS) (n = 1602), disease-specific survival (DSS) (n = 526), and disease-free survival (DFS) (n = 1346) of UCEC is shown.

TMB (tumor mutational burden) and MSI (microsatellite instability) are important biomarkers for immunotherapy and are of clinical practical value, which can evaluate the efficacy of immune checkpoint inhibition therapy in diverse types of cancer [15,16,17]. Therefore, we analyzed the correlation between ABCG2 expression and TMB/MSI across all tumors of the TCGA database using Spearman rank correlation test. As shown in Figure 7C, the expression of ABCG2 in 19 different cancers is significantly correlated with TMB, of which ABCG2 gene expression is positively correlated with a high mutation status in LIHC, LAML, and HNSC, and with a low mutation status in lymphoid neoplasm diffuse large B-cell lymphoma (DLBC), KICH, LUAD, pancreatic adenocarcinoma (PAAD), pheochromocytoma and paraganglioma (PCPG), PRAD, READ, ACC, uveal melanoma (UVM), UCEC, THCA, STAD, SKCM, SARC, BRCA, and CESC. In addition, there are significant correlations between the expression of ABCG2 with MSI in 10 cancer types, including esophageal carcinoma (ESCA), UCEC, THCA, STAD, SARC, LUSC, LUAD, KIRP, DLBC, and BRCA, of which ESCA patients are positively correlated to the expression of ABCG2, and the other cancer types show the opposite trend. Furthermore, we investigated the genetic alteration status of ABCG2 in the different tumor samples of the TCGA cohorts. The types, sites, and case numbers of the ABCG2 genetic alterations are presented in Figure 7A. We found that a missense mutation of ABCG2 was the main type of genetic alteration. For example, the K653E/Y654Ifs*21/K653Nfs*11 alteration in the exon 16 domain was detected in one case of COAD and four cases of UCEC and is displayed in the 3D structure of the ABCG2 protein (Figure 7B). Additionally, we analyzed the potential association between ABCG2 genetic alteration and the clinical survival prognosis in UCEC patients. The data of Figure 8 indicate that UCEC patients with altered ABCG2 show a better prognosis in overall (*p* = 2.452 × 10^−3^), disease-specific survival (*p* = 0.0481), progression-free survival (*p* = 0.0283), and disease-free survival (*p* = 0.0703) compared with patients without ABCG2 alteration.

### 2.5. Analysis of ABCG2-Related Genes and the Correlation between ABCG2 Expression, GSEA, and Checkpoint Gene Expression

**Figure 9:**
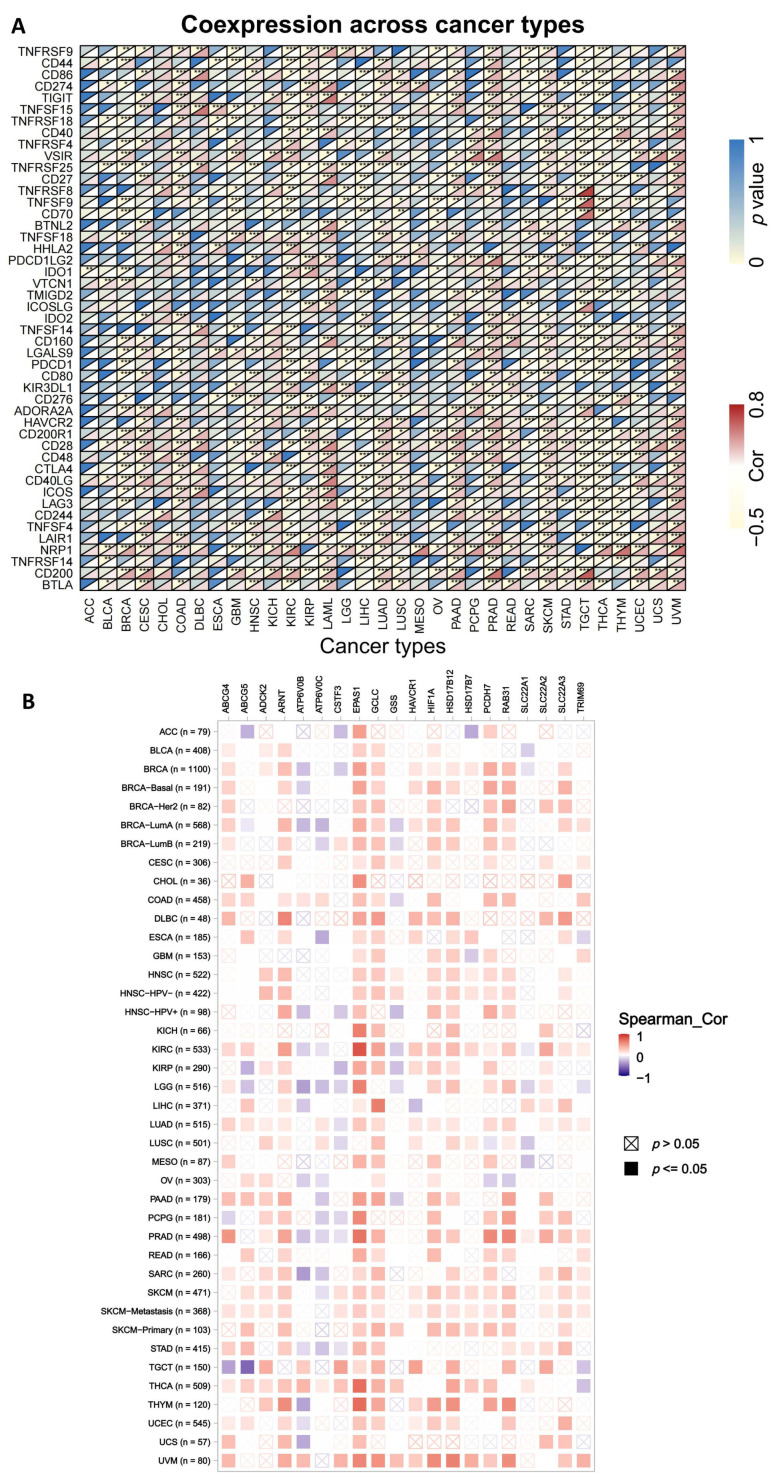
Network of ABCG2-related genes and gene enrichment analysis: (**A**) Co-expression analysis between 47 immune-related genes and ABCG2 gene in the different pan-cancer cohorts of the TCGA database. Upper left corner represents the *p*-value, and the lower right corner represents the correlation coefficient (* *p* < 0.05; ** *p* < 0.01; *** *p* < 0.001). (**B**) The heatmap shows the expression correlation between ABCG2 and the top 20 ABCG2-related genes analyzed by the TIMER2 approach.

The relationship between ABCG2 gene expression and a total of 47 immune checkpoint genes in tumor immune responses was further analyzed. We found that ABCG2 expression shows a close link with T lymphocyte-related immune genes (CD28), T follicular helper cell associated immune genes (CD200 and CD200R1), and cancer-related genes, such as neuropilin-1 (NRP1), tumor necrosis factor receptor superfamily member 25 (TNFRSF25), and galectin-9 (LGALS9), in several different cancer types. Additionally, ABCG2 is significantly co-expressed along with more than 20 immune checkpoint genes in BRCA, CESC, COAD, HNSC, KIRC, KIRP, LAML, LIHC, LUAD, LUSC, PAAD, PRAD, SKCM, TGCT, THCA, THYM, UCEC, and UVM (Figure 9A). These results suggest that the positive association between the expression of ABCG2 and the immune checkpoint genes in various tumors have a function in regulating tumor immune responses.

**Figure 10:**
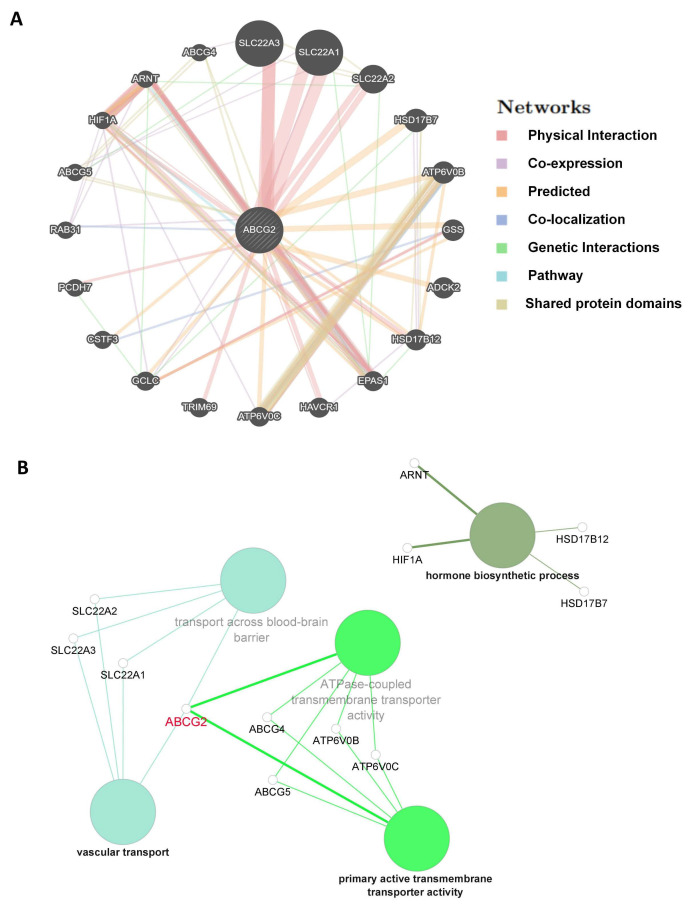
ABCG2-related genes enrichment analysis: (**A**) Protein–protein interaction (PPI) network for the top 20 ABCG2-related proteins based on the GeneMANIA online tool. Different colors of the network edges indicate the bioinformatic methods applied: physical interaction, co-expression, predicted, colocalization, pathway, genetic interaction, and shared protein domains. (**B**) ABCG2 gene and its top 20 related genes were associated to some biological process pathways in GO analysis using Cytoscape. Functionally correlated groups partially overlap and are arbitrarily colored. The node size represents the pathway enrichment significance.

**Figure 11:**
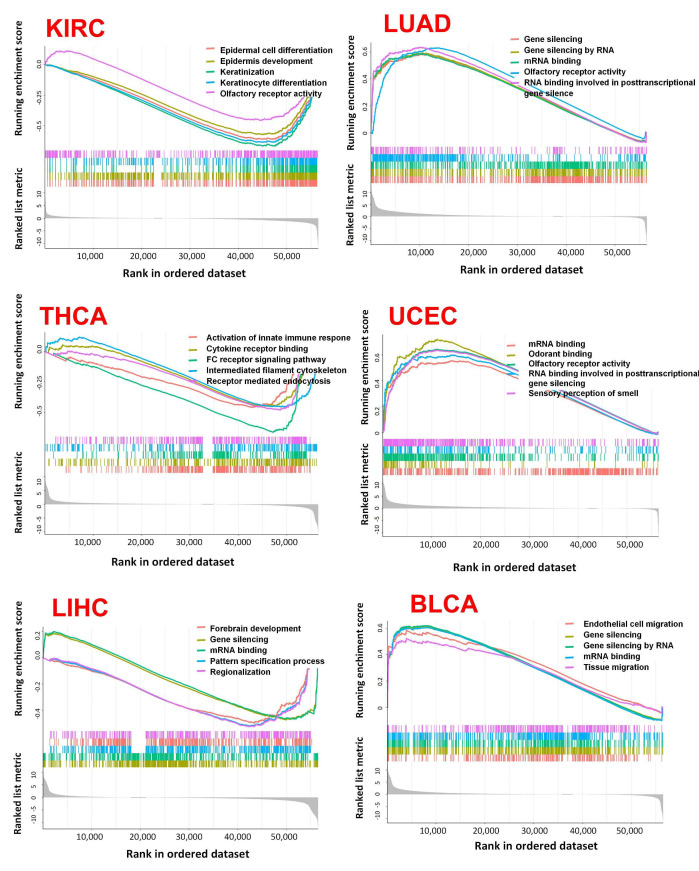
GSEA analysis in six types of cancers. GO functional annotation of the ABCG2 gene in different cancers were displayed. Differently colored curves indicate that the ABCG2 gene regulates different functions or pathways in different cancers. Peaks of curves upward indicate positive regulation and peaks of curves downward represents negative regulation.

The corresponding heatmap data among pan-cancer show a positive correlation between ABCG2 and EPAS1, and GCLC in most cancer types (Figure 9B). The top 20 ABCG2-related genes from the GeneMANIA online tool were analyzed by the protein–protein interaction (PPI) network in Figure 10A. Based on the genes set provided by the GeneMANIA online tool, the ontology (GO) enrichment analysis was used to gather the corresponding pathways among ABCG2 and its 20 related genes, which are vascular transport, primary active transmembrane transporter activity, ATPase-coupled transmembrane transporter activity, transport across the blood–brain barrier, and hormone biosynthetic process (Figure 10B). Furthermore, based on the normalized enrichment scores, we used gene set enrichment analysis (GSEA) that included related genes in humans to find general enrichment trends and to identify GO functional enrichment of different expression levels of ABCG2 among pan-cancer. The most significantly enriched signal transduction pathways were selected, and six types of cancers show the strongest potential connection with the ABCG2 gene expression. The mRNA binding pathway was differentially enriched in BLCA and LUAD with low ABCG2 expression and in LIHC with high ABCG2 expression. Moreover, for high ABCG2 expression, the gene silencing pathway was enriched in UCEC, BLCA, and LUAD, and for low ABCG2 expression, ABCG2 plays an essential role in keratinization progress and epidermis-associated development in KIRC and activation of innate immune response and receptor-mediated signaling pathway in THCA (Figure 11).

### 2.6. ABCG2 mRNA Expression among Six Types of Cancer in Cancer Cells, Cancer Stem Cells, and Corresponding Drug Resistant Cells

**Figure 12:**
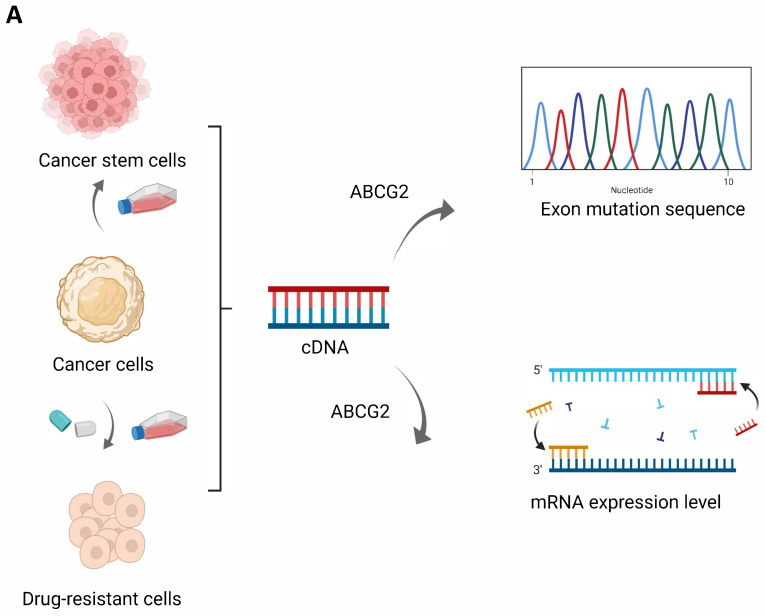

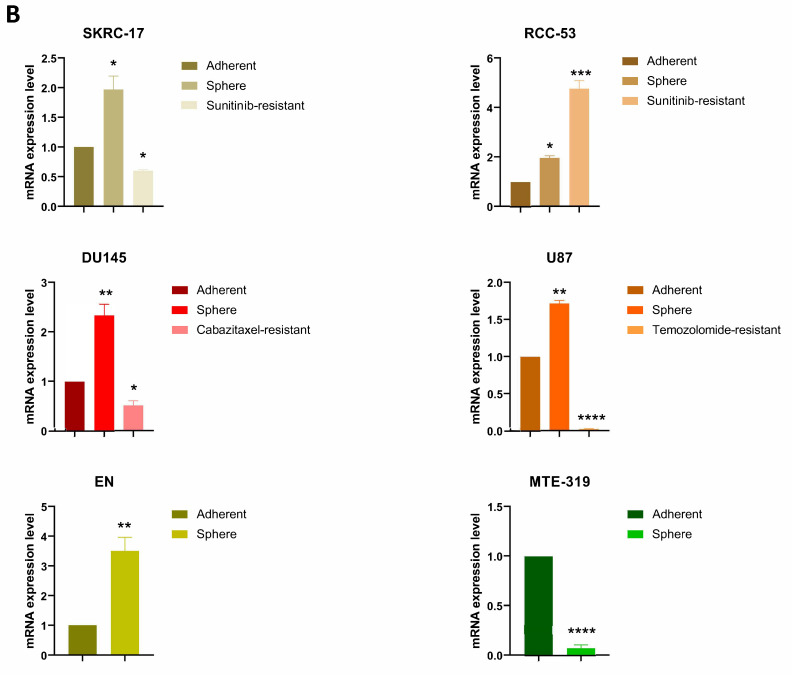
The detection of ABCG2 gene in cancer cells, cancer stem cells, and corresponding drug resistant cells: (**A**) Workflow of cell line preparation and exon sequencing of the ABCG2 gene; (**B**) RT-qPCR analysis representing relative mRNA expression levels of ABCG2 in various cancer cell lines and their corresponding sphere and drug-resistant cell lines. * *p* < 0.05, ** *p* < 0.01, *** *p* < 0.001, **** *p* < 0.0001.

The observed correlation between ABCG2 gene alteration and clinical prognosis in this study and multi-drug resistance function of cancer stem cells inspired us to perform exon sequencing and to analyze the mRNA expression level of ABCG2 among different cancer cells, cancer stem cells, and corresponding drug resistant cells. The cancer cell lines were selected as follows. First, adherent normal cell line, derived sphere-forming cancer stem cells, and sunitinib-resistant cell lines were from the RCC cell lines SKRC-17 and RCC-53 [18]. Sunitinib is the widely used first-line treatment medication for RCC. Second, the prostate cancer cell line-derived spheres [19] and cabacitaxel-resistant cell line are from DU-145 [20]. Cabacitaxel is the second-line treatment option for metastatic castration-resistant prostate cancer. Third, the glioblastoma cell line-derived spheres and temozolomide-resistant cell line are from U87-MG. Temozolomide is the first-line chemotherapeutic drug for glioblastoma. Fourth, adherent and sphere forming cells from the two UCEC cell lines EN (primary endometrial carcinoma, stage IIIC) [21] and MFE-319 (established from the primary adenosquamous endometrium carcinoma, grading G1–G2) were used [22] (Figure 12A). Unfortunately, we could not find any ABCG2 mutations in the exons. For the RT-qPCR analyses (Figure 12B), we used the sphere and drug-resistant cell lines in comparison to the corresponding adherent cell lines. Spheres of all cell lines except MTE-319 express higher levels of ABCG2 mRNA than the adherent cell lines. Drug-resistant cell lines of SKRC-17, DU145, and U87 showed a significantly lower expression level. Only the sunitinib-resistant cell line of RCC-53 showed a significantly higher expression level in comparison to the corresponding sphere and adherent cell lines.

## 3. Discussion

It has been reported that ABCG2 plays multifunctional roles in human diseases, such as inducing multidrug resistance in cancer treatment, contributing to the risk for hyperuricemia and gout, and overexpressing in resistant acute myeloid leukemia phenotype [23,24,25,26,27,28]. Emerging publications have reported a functional link between ABCG2 as well as its genetic alterations and the clinical relevance in tumors [29,30]. Whether ABCG2 participates in the pathogenesis or pharmacokinetics in all types of cancer through certain common molecular mechanisms remains to be clarified. A literature search failed to retrieve any publication with a pan-cancer analysis for ABCG2. Thus, we comprehensively examined the ABCG2 gene expression in a total of 33 different tumors based on the TCGA, CPTAC, and GEO databases and analyzed the molecular features of gene expression, prognosis, tumor microenvironment, tumor stemness, and genetic alteration to further explore the multifunctional role of ABCG2 gene in different cancer types.

Based on the results, ABCG2 was differentially expressed in tumors, of which a lower expression was found in most cancer types but a significantly higher expression in KIRC and LGG patients, which illustrated the flexible function of ABCG2 among pan-cancer. For normal healthy tissue expression groups, the ABCG2 can be regarded as an essential protector from xenobiotics as an oral bioavailability regulator, part of component of the blood–brain barrier, the blood–testis barrier, and the maternal–fetal barrier [31]. In contrast, in ABCG2 high expressing cancer tissues, ABCG2 contributes to drug resistance in cancer treatments. Moreover, previous research showed that two SNPs in ABCG2 have a significant correlation with mRNA expression in ccRCC tissues in univariate analyses (mRNA: rs76212402, rs2231164), but no significant association was found for BCRP/ABCG2 protein expression [32]. Thus, the potential of ABCG2 as therapeutic target to overcome multi-drug resistance strategies by ABC drug transporter inhibitors to increase the efficiency of chemotherapy should be further discussed.

ABCG2 is considered as a biomarker of cancer stem-like cells (CSCs), which can arise from various sources, including long-lived stem or progenitor cells or via dedifferentiation from non-stem cancer cells. The capacity for self-renewal and invasion of CSCs can promote cancer progression, which is the main cause for treatment-induced drug resistance [33,34,35]. In the present study, we investigated the association of ABCG2 with stem cell-like features using the RNAss and DNAss scores [36]. Unexpectedly, we found that ABCG2 expression was negatively associated with cancer stem-like features, RNAss among most types of cancers. We assume that the reason for this trend could be related to the tumor purity among pan-cancer samples. Apart from the tumor cells, the tumor tissues in the TCGA database also consist of other cell types, such as stromal and immune cells, which means that tumor purity could be a potential influencing factor on ABCG2 expression and the stemness scores. By contrast, we can see from the results of evaluating the stromal and immune scores in KIRC samples, ABCG2 expression significantly shows an opposite tendency, suggesting that ABCG2 plays a role in the KIRC tumor microenvironment and can be used as a therapeutic target to aid drug sensitivity.

Several studies have reported a role of ABCG2 in the progression or prognosis of renal cell carcinoma [13,32,37]. In 2017, Wang et al. reported that stronger ABCG2 expression correlates with poorer overall survival. The median overall survival was 93.2, 85.8, and 17.0 months in ABCG2(−), (+), and (++) subgroups, respectively, and the five-year survival rate was 95%, 77%, and 27% in ABCG2(−), (+), and (++) subgroups, respectively [13]. However, we found that low expression of ABCG2 was associated with poorer overall survival and higher grades of RCC. The reason for this discrepancy is as follows: First, it should be noted that no more than 60 cases of RCC patients were included in either ABCG2(−), (+), and (++) subgroups in Wang’s study; thus, the different sample sizes perhaps are warranted for verification of the above conclusion. Second, the significant associations for ccRCC patients with survival were found for the ABCG2 SNPs rs2622621 and rs3109823 in univariate analyses [32], so that the additional patients’ features should be fully considered as well. Third, 101 patients without metastasis were treated for one year with interferon-alpha in Wang’s study, and 19 patients with metastatic RCC received Sorafenib or Sunitinib. However, because of the tremendous data of the pan-cancer study, we did not include the treatment features of patients yet; thus, more clinical details of the patients should be considered in the following investigations for RCC.

In addition, more interesting results were found for the correlation between ABCG2 gene alteration and prognosis in this study. First, we discovered a significant difference between ABCG2 gene alterations and ABCG2 wild type regarding OS, PFS, and DSS in UCEC patients. As we mentioned before, ABCG2 mutations can also alter the survival progress in renal cell carcinoma patients [32]. Moreover, investigations of the correlation of ABCG2 gene mutations with ABCG2 transport activities and further clinical pharmacogenetic studies have also been carried out in various types of cancer [38,39,40,41,42]. One of the main reasons for the association between gene alteration and clinical features may lie in the nature of the substrate transport process. ABCG2 is a symmetrical homodimer consisting of two proteins, each with a nucleotide binding domain (NBD) and a transmembrane domain (TMD) building the substrate binding site. When a substrate binds to the binding pocket, NBDs face away from each other and ATP binds, thus inducing a conformational change. The substrate is released from the protein. When the second ATP molecule is hydrolyzed, the conformation is reset and substrate and ATP can rebind, allowing the process to repeat. The study from Manolaridis et al. found that the human ABCG2 gene with the mutation E211Q can drastically reduce the catalytic activity for ATP hydrolysis within the NBD. This mutation was helpful to trap the protein conformation in the respective state [43]. Thus, the mutations can change the transport efficiency and, therefore, the pharmacokinetics, which may be reflected in the patient’s clinic. The development of an endometrial carcinoma starts with preinvasive intraepithelial lesions and estrogen stimulation and then penetrates into the myometrium to reach lymphatic capillaries and the regional lymph nodes from where metastases reach other organs through vascular channels [44]. Using the GO enrichment analysis (Figure 10B), we know which of the ABCG2-related genes are associated with the “vascular transport” and “hormone biosynthetic process”. Thus, we can speculate that the ABCG2 gene plays an essential role in the pathogenesis and the pharmacokinetics in UCEC patients. More in-depth molecular experiments are needed to verify these results.

The observed correlation between ABCG2 gene alteration and clinical prognosis in this study and the emerging publications of the resulting multi-drug resistance inspired us to analyze the correlation of ABCG2 gene alteration and mRNA expression level among different cancer cells, cancer stem cells, and corresponding drug resistant cells. Based on the sequencing results, we could not find any ABCG2 mutations in the exons. Further experiments, such as intron sequencing and protein structure analysis, will follow in order to be able to analyze correlations between ABCG2 gene mutations and therapy resistance. For the qPCR result, sphere groups had a higher expression level of ABCG2 compared to the adherent group in DU145 cell lines, which were similar to the results from the former research’s results [20]. The significantly different expression levels of the ABCG2 gene between drug-resistance groups and the adherent groups were explored in this article for the first time, which implied that ABCG2 acts multi-functionally in the different types of drug-resistant cancer cells. Moreover, the higher expression of ABCG2 in spheres and sunitinib-resistant cell lines of RCC-53 compared to the adherent cell line, showed that further in-depth research, such as analyzing tumorigenicity of different ABCG2 expressing sub-phenotypes in RCC-53 cell lines, can be organized in the future. Furthermore, endometrial carcinoma cell lines were selected in this part of the experiments not only because of impressive results observed in our study but also because ABCG2, as one of ATP-binding cassette genes, is involved in estrogen transport; meanwhile the increased estrogen action is closely associated with endometrial carcinoma [45]. Based on our result, the different expression levels observed between adherent and sphere cell lines from endometrial carcinoma can be the result of the category of cancer type. MTE-319 is established from primary adenosquamous endometrial carcinoma containing both malignant glandular and malignant squamous components [46], and EN is established from stage IIIC of primary endometrium carcinoma, showing that ABCG2 gene expression probably displays various stemness capacity in different types of endometrial carcinoma.

In this study, for the first time, a pan-cancer analysis of ABCG2 expression and its correlation with clinical prognosis, stemness score, tumor microenvironmental score, TMB, and MSI was performed, aiming to understand the role of ABCG2 in tumorigenesis. Furthermore, the results from the correlation between ABCG2 mutations and clinical prognosis helps to realize personalized medicine. More clinically relevant mutations in membrane proteins on tumor cells should be analyzed in the future.

## 4. Materials and Methods

### 4.1. Gene Expression Analysis

We loaded ABCG2 into the “Gene_DE” module of TIMER2.0 (tumor immune estimation resource, version 2; (http://timer.cistrome.org/) (accessed on 19 June 2022), which can provide four modules for investigating the correlations between immune infiltrates and genetic or clinical features and four modules for exploring cancer-related associations in the TCGA cohorts [47] and analyze the expression patterns of ABCG2 between tumor and adjacent normal tissues for the different tumors or specific tumor subtypes of the TCGA project.

For certain tumors without normal or with highly limited normal tissues, we used the “expression analysis-box plots” module of the GEPIA2 (Gene Expression Profiling Interactive Analysis, version 2) web server (http://gepia2.cancer-pku.cn/#analysis) (accessed on 19 June 2022) to obtain box plots of the expression difference between these tumor tissues and the corresponding normal tissues of the GTEx (Genotype-Tissue Expression) database [48], under the settings p-value cutoff = 0.01, log2FC (fold change) cutoff = 1, and “Match TCGA normal and GTEx data”.

The UALCAN portal (http://ualcan.path.uab.edu/analysis-prot.html), an interactive web resource for analyzing cancer omics data, allowed us to conduct protein expression analysis of the CPTAC (clinical proteomic tumor analysis consortium) dataset [49] (accessed on 19 June 2022). Herein, we explored the expression level of the total protein of ABCG2 between primary tumor and normal tissues, respectively, by entering “ABCG2”. The available datasets of three tumors were selected, namely, clear cell RCC (renal cell carcinoma), UCEC (uterine corpus endometrial carcinoma), and LUAD (lung adenocarcinoma).

Immunohistochemistry (IHC)-based protein expression images of ABCG2 protein expression in clinical specimens of cancer patients were obtained from the Human Protein Atlas database (https://www.proteinatlas.org/) (accessed on 6 September 2022) [50].

### 4.2. Prognosis Analysis

To investigate the prognostic value of ABCG2 in pan-cancer, we extracted patient data, including OS (overall survival), DFS (disease-free survival), and other clinical features with detailed follow-up and survival information from the TCGA database. The data were first analyzed by “survival” R package (https://www.r-project.org/) (accessed on 17 May 2021), and then the survival maps were displayed in GraphPad Prism 7.0. The Kaplan–Meier curve of ABCG2 in the different cancers were plotted using the “ggplot2” package, where *p* < 0.05 was shown. Moreover, the correlation between patient stage, age, gender, and ABCG2 expression among the different cancer types were analyzed by the R packages “ggpubr” and “limma”.

### 4.3. Tumor Stemness Indices and Tumor Microenvironment Scores

According to the transcriptome and to the epigenetic characteristics of TCGA tumor samples, the stem cell-like features of tumor cells were detected. The correlation of cancer stemness with ABCG2 expression was evaluated using Spearman correlation test. Furthermore, the degree of infiltration of immune and stromal cells in the distinct tumor tissues was analyzed based on the RNA-seq data from the TCGA database (accessed on 18 March 2021) in the “estimate” and “limma” R package and displayed in “ggplot2”, “ggpubr”, and “ggExtra”R packages. The score maps were performed using GraphPad Prism 7.0.

### 4.4. Genetic Alteration Analysis

Tumor mutational burden (TMB) was evaluated based on Perl scripts, and this value was corrected by dividing by the exon length. Microsatellite instability (MSI) scores were extracted using TCGA (accessed on 3 May 2021). The correlation between ABCG2 expression and either TMB or MSI was analyzed using the “cor.test” command based on Spearman’s method. The two metrics were visualized using radar plots, which were generated using the R package “fmsb”.

The cBioPortal web (https://www.cbioportal.org/) (accessed on 10 January 2022) was used to analyze the ABCG2 gene alteration analysis, The results of the alteration frequency across all TCGA tumors were analyzed in the “Cancer Types Summary” module in the “TCGA Pan-Cancer Atlas Studies”. The schematic diagram of the protein structure and the mutated sites in the 3D (three-dimensional) structure of ABCG2 can be operated via the “Mutations” module. Then, we selected the three studies: Uterine Corpus Endometrial Carcinoma (TCGA, Firehose Legacy) (549 samples), (TCGA, Nature 2013) (373 samples), and (TCGA, Pan-cancer Atlas) (529 samples) to generate the clinical data. The “Comparison” module was used to obtain the data for overall survival (OS), progression-free survival (PFS), disease-free survival (DFS), and disease-specific survival (DSS) differences with or without ABCG2 genetic alterations, and Kaplan–Meier plots with log-rank *p*-values were generated as well.

### 4.5. Co-Expression of ABCG2 with Immune-Related Genes, ABCG2-Related Genes, and Pathways in Tumors

The R packages “limma”, “reshape2”, and “RColorBrewer” were used for analyzing the co-expression with immune-related genes.

GeneMANIA online database tool (http://www.genemania.org) (accessed on 10 January 2022) was applied for ABCG2-related gene analysis and its protein–protein interaction (PPI) analysis, which includes physical interaction, co-expression, co-localization, gene enrichment analysis, genetic interaction, and website prediction [51].

The software Cytoscape (version 3.8.2) (https://cytoscape.org/) (accessed on 17 April 2022) and its plug-in ClueGO were used to perform the ABCG2-related genes’ pathway enrichment analysis in gene ontology (GO)-biological process. *p* < 0.05 and minimum gene numbers, which must be more than four, were set up [52,53].

The “Gene_Corr” module of TIMER2.0 (accessed on 10 January 2022) was used to supply the heatmap data of the selected genes, which contains the partial correlation (cor) and *p*-value in the purity-adjusted Spearman’s rank correlation test.

Gene ontology (GO) gene sets were obtained from the Gene Set Enrichment Analysis website (GSEA, https://www.gsea-msigdb.org/gsea/downloads.jsp) (accessed on 1 April 2020). GO functional annotations and enriched pathways associated with ABCG2 expression were analyzed using the R packages “limma”, “org. Hs.eg.db”, “clusterProfiler”, and “enrichplot”.

### 4.6. Cell Culture

SKRC-17 (kind gift from J. Vissers, Nijmegen), RCC-53 (derived from a patient with stage IV disease (pT2N1MxG2-3)), sunitinib-resistant SKRC-17 and RCC-53 [18], DU145, cabacitaxel-resistant DU-145 [19,20], U87-MG (from European Collection of Authenticated Cell Cultures), temozolomide-resistant U87-MG, and MFE-319 (established from the primary adenosquamous endometrium carcinoma, grading G1–G2) [22] were cultured in RPMI 1640 medium, and EN (primary endometrial carcinoma, stage IIIC) [21] was cultured in DMEM GlutaMAX medium, both supplemented with 10% fetal calf serum (FCS “Gold Plus”, Bio & Sell GmbH, Feucht, Germany), 2 mM L-glutamine, 1 mM sodium pyruvate, and 1% minimal essential medium (Invitrogen, Life Technologies GmbH, Darmstadt, Germany) at 37 °C in humidified incubator with 5% CO_2_. CSCs were cultured in DMEM/F12 culture medium, containing 2% B-27 (Invitrogen, Life Technologies, Darmstadt, Germany) 10 ng/mL human recombinant basic fibroblast growth factor (bFGF, Sigma Aldrich Chemie GmbH, Taufkirchen, Germany), and 10 ng/mL epidermal growth factor (EGF, Sigma Aldrich).

### 4.7. Sphere Formation Assay

SKRC-17, RCC-53, DU145, U87-MG, EN, and MFE-319 adherent cell lines were collected by StemPro^®^ Accutase^®^ (Life Technologies, Thermo Fisher Scientific, Waltham, MA, USA). Then, harvested adherent cells were filtered through a cell strainer with 40 µM nylon mesh (BD Biosciences, Heidelberg, Germany) and seeded in 75 cm^2^ ultra-low attachment flasks (Corning, New York, NY, USA) with 10 mL serum-free CSC medium. After seven days, the spheres were harvested and dissociated by Accutase for 10 min at 37 °C. After being centrifuged at 500× *g* for 4 min at room temperature, cells were collected and used for the following assays.

### 4.8. RNA Extraction, Reverse Transcription, and Sequencing

Total RNA was extracted from cells using the RNeasy Mini-Kit (Qiagen, Hilden, Germany) based on the manufacturer’s instructions. The cDNA was synthesized according to kit instructions (GoScript™ Reverse Transcriptase System, Promega GmbH, Walldorf, Germany). ABCG2 mRNA sequencing was performed by the company Eurofins Genomics (Ebersberg, Germany).

### 4.9. Quantitative Reverse Transcription PCR (RT-qPCR)

RT-qPCR was performed using the FastStart Essential DNA Green Master kit (Roche, Penzberg, Germany) and the LightCycler^®^ 96 (Roche). Data were analyzed by the LightCycler^®^ 96 software version 1.1. The relative expression analysis was carried out by the 2^−ΔΔCt^ method. The annealing temperature was 60 °C for all primers, listed in Table 1, and the transcription level of GAPDH and ACTB was used as an internal control.

## Figures and Tables

**Table 1:** Primers used for RT-qPCR.

Transcript	Primer	Sequence (5′–3′)	Product Size (bp)
GAPDH	GAPDH-F	CATGGGTGTGAACCATGA	104
	GAPDH-R	TGTCATGGATGACCTTGG	
ACTB	ACTB-F	CTGCCCTGAGGCACTC	197
	ACTB-R	GTGCCAGGGCAGTGAT	
ABCG2	ABCG2-f	CATCAACTTTCCGGGGGTGA	266
	ABCG2-r	CACTGGTTGGTCGTCAGGAA	

## Data Availability

The raw data supporting the conclusions of this article will be made available by the authors, without undue reservation.
